# On Inflammation, Chronic Disease, and Perforative Ulceration of the Cæcum, and of the Appendix Vermiformis Cæci, with Symptomatic Peritonitis and Fæcal Abscess

**Published:** 1840-04

**Authors:** 


### ART. VIII.

1. *On Inflammation, Chronic Disease, and Perforative Ulceration of the Cæcum, and of the Appendix Vermiformis Cæci, with Symptomatic Peritonitis and Fæcal Abscess.* By JOHN BURNE, M.D., Physician to the Westminster Hospital, (*Med. Chir. Trans.*, Second Series, Vol. II.—*London*, 1837.)

2. *Memoir on Tuphlo-Enteritis; or Inflammation and Perforative Ulceration of the Cæcum, and of the Appendix Vermiformis Cæci.*—By JOHN BURNE, M.D., Physician to the Westminster Hospital. (*Med. Chir. Trans.*, Second Series, Vol. IV.—*London*, 1839.)

3. *Beobachtungen auf dem Gebiete der Pathologie und Pathologischen Anatomie.* Von Dr. J. F. ALBERS.—*Bonn*, 1838. 8vo, pp. 218.
*Observations in Pathology and Pathological Anatomy.* (*Chapter on Typhlitis.*) By Dr. J. F. ALBERS.

4. *Histoire de Tumeurs Phlegmoneuses de Fosses Iliaques.* Par le Docteur GRISOLLE, Ancien Chef de Clinique à l'Hôtel-Dieu, &c. (*Archives Génerales de Médecine, pour Janvier, Fevrier, et Mars.*—*Paris*, 1839.)
*History of Phlegmonous Tumours of the Iliac Fossæ.* By Dr. GRISOLLE.

IN the preceding article, in which we have given an analysis of all the more important papers in the last volume of the Medico-Chirurgical Transactions, we passed over entirely the valuable memoir of Dr. Burne on *Tuphlo-Enteritis*, or Inflammation of the Cæcum. We did so because we wished to consider the subject of that paper at greater length than we could then conveniently do, and to comprehend in our notice not only a former memoir of Dr. Burne's, but also some publications on the same affection which have recently appeared on the continent. To this purpose, then, we devote the present article, to which we shall give as much of a practical character as possible; well knowing that the diseases under discussion are more common than is generally believed, and are but imperfectly understood by many practitioners, to whom they are not unfamiliar.

An accumulation of fæces in the bowels, sufficiently great to give rise to a perceptible tumour, and usually accompanied by constipation, and more or less of local pain, is a case well known to all practical men. This accumulation may exist anywhere in the course of the large intestine. After the patient has been costive for some days, he is seized with griping pain in some part of the abdomen; and, on examination, a well-defined tumour is discovered, which generally offers some unevenness of surface. At first there is no fever, and but little constitutional disturbance, except some degree of listlessness; but the expression is dull, the tongue is covered with a moist white coat, and indented, and the appetite is impaired. On the free action of a purgative, all these symptoms are removed, and the patient is well. If this remedy be not had recourse to, or if its action be insufficient to remove the obstruction, the collection which forms the tumour increases, and by distending the bowels, occasions the most exquisite tenderness; the patient shivers and becomes feverish; and, occasionally, vomiting and all the symptoms of ileus occur. In some cases, the obstruction is not complete, the bowels act perhaps daily, and from a superficial enquiry, the physician would be led into error as to the nature of the tumour; but, on pursuing his investigation, he will find that the evacuations, though regular, are very scanty and liquid.

Although a collection of fæces, sufficient to form a perceptible tumour, may occur in any part of the large intestine, the most frequent seat of such tumours is the cæcum. This is explained by the pouch-like form of the cæcum, by its being bound down in the right iliac fossa, and consequently admitting of less motion than other parts of the large intestine, and by the circumstance that its contents, which have acquired the excrementitial character, are obliged to move in a vertical direction, or against the force of gravity. It is to cases of this kind, that is, to cases in which the cæcum is distended by a collection of fecal matter, and its mucous membrane in consequence inflamed, that Dr. Burne has given the name of "*Tuphlo-Enteritis.*"

But other tumours occasionally form in the right iliac fossa, in dependence on the cæcum, which, with reference both to prognosis and treatment, we must carefully distinguish from the other class of cases. Dupuytren, in his "Leçons Orales de Clinique Chirurgicale," speaking of abscesses in the right iliac fossa, says, "I have long taught that tumours form in the right iliac fossa, which seem intimately connected with the parietes of the cæcum. These tumours are frequently attended by remarkable disturbance in the function of the large intestine: in a great number of cases they terminate by resolution; in some circumstances by abundant suppuration; and sometimes they are the origin of a general peritonitis." (Tom. iii., p. 331.) In this passage Dupuytren has well pointed out the different terminations of tumours that are met with in the right iliac fossa in connexion with the cæcum. He also remarked that such tumours are preceded or attended by different trains of symptoms —sometimes by diarrhœa, at other times by constipation. But, notwithstanding these differences, he supposed them to be all of the same kind, namely, all tumours formed by the engorgement of the cellular tissue in which the cæcum is imbedded. He held, in accordance with the doctrines of Broussais, that these engorgements may result alike from any cause that produces irritation of the mucous membrane of the neighbouring portion of the large intestine; and that they sometimes terminate by suppuration, but more commonly by resolution. He seems to have never once suspected that the tumour might be simply a collection of fæces.

Dr. Burne has, in the papers before us, rendered a valuable service to pathology, by showing that these tumours differ essentially in kind, and by pointing out the characters by which their different varieties may in general be distinguished. In proceeding to give some analysis of the cases detailed in these papers, we shall divide them into two classes: the 1st including those in which we imagine the tumour to have resulted from an accumulation of fæces; the 2d, those in which it arose from a collection of pus.

1. *From Fecal Distension.* By far the majority of cases of tumour in the right iliac fossa belong to the first category. As an example of them we may cite the second case given by Dr. Burne:—A man is found complaining of extreme pain and tenderness in the situation of the cæcum, where there is discovered "a hard body, like something lodged in the blind extremity of that gut." The bowels had been constipated for a week. He was freely bled and leeched, but the issue seemed doubtful, when the bowels began to act copiously, and the patient recovered. (*Med. Chir. Trans.*, vol. xx., p. 210.)

Occasionally, the obstruction is more complete; and, besides the local pain and tenderness, the patient has vomiting and other symptoms of obstacle to the passage of fæces. The first case of Dr. Burne is a fair specimen of such cases.

A boy, twelve years old, was seized one morning with aching pain in the right ilio-inguinal region. The pain increased as the day advanced; it did not remit or recur in paroxysms, but remained fixed and constant. On the morrow he began to be feverish and to vomit. On the third day leeches were applied to the seat of the tumour, and aperients were given. On the fourth day, the symptoms increasing, blood was drawn to the extent of fifteen ounces, and strong aperient medicines were continued. On the fifth day more leeches were applied. He was then seen for the first time by Dr. B., who thus describes his condition. "The boy was lying on his back quite still, careful not to move his body lest it should aggravate the pain: the whole abdomen was tumid and tense, more particularly in the right ilio-inguinal region, where there was a fixed aching pain, with tenderness so excessive that the slightest touch was reluctantly borne, while over the other regions of the abdomen the tenderness and pain were trifling: he was sick and vomited from time to time; the tongue was foul and brownish; the pulse frequent and weak; there had been no action of the bowels since the commencement of the illness, now six days, except from the colon by an enema on the second day." (*Med. Chir. Trans.*, vol. xx., p. 209.) Dr. Burne recommended fomentations and gentle saline aperients. On the following day, the bowels had acted several times; some hard lumpy matter had been discharged; and the tenderness, pain, and tension had diminished. From this time the boy's health was quickly and perfectly reestablished.

Instances of the same kind are so frequent among the lower orders of this country, who disregard the slighter inconveniences of constipation, that all medical men, who have much experience in hospital or dispensary practice, must be very familiar with them. We could add a long list to the cases given by Dr. Burne, but the task is needless, as their general characters are very uniform; the cases become more alarming in proportion as the obstruction is more complete, or more difficult to remove. Cases 9 and 10 of Dr. B., come under the category we are now considering; and the last of them is worthy of notice, as showing how difficult it sometimes is to remove these accumulations of fæces. The patient was a gentleman of middle age, and of full habit and stature.

"He was ill in bed, with pain, great tenderness, and a deep-seated resisting circumscribed fulness, amounting almost to a tumour, in the right ilio-inguinal region; the rest of the abdomen was full and slightly tender, but free from pain; the bowels had been obstinately confined for several days, and sickness and vomiting were frequent; the tongue was very much loaded, the urine red with copious deposits of the lithates and purpurates, and the skin of a feverish heat; the pulse was frequent, rather large, but compressible. He had been bled and leeched, had used a hot-bath, and taken calomel and purgatives. The countenance was anxious, and he was much exhausted from several days' suffering, as well as from the necessary antiphlogistic treatment. The disease was manifestly an inflammation of the cæcum. Calomel and opium, with the saline effervescing aperient every four hours, were prescribed: the part to be frequently fomented. The symptoms persisted for two days, when evacuations took place, and he began to recover. The evacuations, though kept up afterwards without difficulty, were insufficient; and there remained the same deep-seated circumscribed resistance in the region of the cæcum, with fulness and flatulence of the belly, and a sensation to the patient that he wanted to be more freely purged, the bowels seeming to him to act only to a certain point, from which the flatus, instead of passing on, would roll back in the intestines. On this account he took a brisk cathartic, composed of colocynth and calomel, followed by senna and salts. These, instead of producing relief, brought on a return of sickness, an increase of the fulness, and of the pain in the cæcal region, and induced us to return to the more mild and appropriate saline aperients, under the use of which the unfavorable symptoms again subsided. His health, however, did not forthwith return; the tongue remained foul, the appetite defective, and the circumscribed fulness was still to be felt in the region of the cæcum. To resolve this, mercurials were persevered in, and it was not till after five or six weeks of strict regimen, and the daily use of a saline aperient, that all evidence of the local disease passed away, and recovery was reestablished." (*Med. Chir. Trans.*, vol. xxii., p. 38.)

In all these cases, the inflammation of the cæcum was evidently secondary, the consequence of some mechanical irritation; and the violent symptoms uniformly subsided when the bowels acted freely. In such instances, when the nature of the tumour is well ascertained, the use of purgatives must be continued; and we agree with Dr. Burne in thinking that saline aperients, in small doses are the best.

Dr. Burne states that an idiopathic inflammation of the cæcum from the usually assigned cause, exposure to vicissitudes of weather, has never fallen under his notice. On this point, our own experience agrees completely with that of Dr. Burne. We have never met with a case of idiopathic inflammation, affecting the cæcum only. Ulcers are, indeed, often found in the cæcum in persons who have died of dysentery; but they are, we believe, never limited to the cæcum, nor do they often exist in the highest degree in that portion of the large intestine.

Dr. Albers has, however, devoted a chapter of his essay to what he calls "typhlitis acuta"—acute idiopathic inflammation of the cæcum. It appears to us, from the description which he has given of this affection, that he has confounded under this title cases of dysentery, in which the cæcum was affected together with the rest of the large intestine, and cases in which the cæcum was inflamed in consequence of its distension by a collection of fæces. As symptoms of this idiopathic inflammation of the cæcum, (which he says is not a rare affection,) Dr. Albers enumerates—a constant, uniform, burning pain in the right iliac fossa, which sometimes extends in the direction of the ascending and transverse colon; tension and *hardness* in the same part; and pain and *numbness* extending from the right side of the sacrum towards the right thigh. The *hardness* which is felt in the cæcal region Dr. Albers supposes to be caused by the inflamed portion of intestine; and the pain and *numbness*, by the *pressure* which this inflamed portion exerts upon the muscles situated beneath it. We can hardly conceive that the coats of the intestine can be so thickened and indurated, by acute inflammation, as to cause the circumscribed hardness which Dr. Albers has noticed; or that they could become so heavy as to produce, by their pressure, pain and numbness of the thigh. In a subsequent part of his paper, when speaking of inflammation of the cæcum produced by an accumulation of fæces, (typhlitis stercoralis,) Dr. Albers lays great stress on the very same symptoms, and especially on the painful numbness of the right thigh and pelvis, caused by pressure on the internal iliac muscle. We believe, then, that the cases which Dr. Albers supposed to be cases of idiopathic inflammation of the cæcum were, like those we have already cited from the papers of Dr. Burne, cases in which inflammation of the cæcum was excited by an accumulation of fecal matter; and we are led to this belief the more readily, as Dr. Albers has not substantiated his opinion by the details of the very cases recorded by him. In the chapter which he has devoted to acute inflammation of the cæcum, he has given the particulars of only two cases; and these are very unhappily chosen for the maintenance of the opinion, that acute idiopathic inflammation of the cæcum is a common disease; the first being one, in which inflammation of the cæcum was caused by its strangulation in a hernial sac; the second, quoted from the Sepulchretum of Bonetus, that of a man who had swallowed 800 pins, and at the opening of whose body "the cæcum was found in suppuration."

In all the cases to which we have as yet referred, the collection of fæces has been in some sort accidental; it has formed in persons who perhaps have experienced slighter degrees of constipation, but whose bowels have been in other respects healthy. In all such cases, removal of the obstruction is followed by complete recovery of the patient.

Occasionally, however, the obstruction is the result of some organic disease, which impedes or entirely prevents the natural movements of the part. The cæcum, as well as the pyloric extremity of the stomach and the rectum, is liable to a chronic disease affecting chiefly the submucous cellular tissue, which becomes hypertrophied and almost cartilaginous in structure, and which causes contraction of the natural passage, and, finally, all the evils which result from its obstruction. The disease, when seated at the rectum, may be considerably relieved by the use of bougies, which produce a mechanical dilatation of the contracted part; but when the pylorus or the cæcum is thus affected, the malady admits of little palliation, except from attention to diet. When a collection of fæces forms in the cæcum from such a cause, its removal (which is extremely difficult to effect) will be followed by the relief of urgent symptoms; but, of course, we cannot hope for eventual success. The third and fourth cases of Dr. Burne offer an illustration of the preceding remarks.

The third case is that of a woman, aged thirty-one, who was admitted into Guy's Hospital in a very emaciated state, from an illness which had afflicted her many months, and of which the prominent symptoms were an obstinately constipated state of the bowels, together with sickness so frequent that the greater part of her food was cast up again. She complained of pains in the belly, like the throes of labour; and in the right ilio-inguinal region a small hard tumour was discovered, which was supposed to be the cæcum diseased; the abdomen was tumid and flatulent, and a strong vermicular motion within the belly was observable, corresponding exactly with the peristaltic action of the intestine; besides which, the distended convolutions of the small intestines could also be accurately traced by the undulating elevations of the abdominal parietes. Various means were employed without benefit, and in the course of a fortnight she died. On dissection, the cæcum was found thickened and much contracted, the ileum distended with flatus, and loaded with feculent matter, while the colon was empty and contracted. (*Med. Chir. Trans.*, vol. xx., p. 212.)

The subject of the fourth case was a girl, twelve years old, very small in stature, but not deformed. Ill health, it was said, had impeded her growth for several years. The history of her complaints, as far as could be ascertained, was that she had suffered for two or three years from large tumid abdomen, with a very irregular and difficult state of the bowels, flatulence, spasmodic pains, loss of appetite, continued emaciation, and frequent attacks of sickness and vomiting. The dejections were generally scanty, soft, and very offensive. A fortnight before her death, she was seized with unusually severe pain in the belly, followed quickly by sickness and vomiting: the abdomen was immensely distended, and very tender; and the dejections consisted of mucus only, without any trace of feculent matter. The vomiting continued till her death, prior to which, for some days, the matter thrown up was distinctly feculent. The body, which was extremely emaciated, was examined fourteen hours after death. When the abdomen was opened, the small intestines were seen distended excessively, being three or four inches in diameter, of a mottled livid red, and agglutinated by soft albuminous matter. Tracing the intestinal canal, the seat of the obstruction was found at the cæcum, to which point the distension of the bowels continued, while beyond it the colon was empty, contracted, and sound. The cæcum when removed and examined proved to be contracted and thickened, its tunics being blended together, and transformed into a dense, opaque, white, unyielding, gristly substance; and anteriorly were discovered numerous organized bands covered with a smooth shining membrane, stretching across the channel of the gut from side to side, in various directions, forming an irregular coarse network. In this contracted cæcum and network, a complete obstruction had been formed by feculent matter, dry and friable, plugging up the channel in a manner which nothing could have removed. Leading to this part was the ileum, filled with an amazing quantity of soft, yellow, homogeneous feculent matter, the bowel itself having all its tunics confounded together, and being converted into a dense strong tissue, a line in thickness, resembling thick wet parchment; all trace of villous structure, or valvulæ conniventes, having disappeared. The colon was healthy. (*Med. Chir. Trans.*, vol. xx., p. 213.)

The forms of disease witnessed in these two cases, especially the latter in which small organized bands stretched across the channel of the gut, are of frequent occurrence in the rectum, and are mentioned by authors as the cause of those varieties of stricture of the rectum, which are benefited in the most decided manner by the use of bougies.

II. *From a Collection of Pus.* The cases included in the second category, those, namely, in which the tumour in the iliac fossa is formed by a collection of pus, differ materially from the preceding. We have already stated that Dupuytren supposed the abscess in these cases to be seated outside the peritoneum in the cellular tissue behind the cæcum, and to be the result of irritation of the mucous membrane of that portion of intestine. Dr. Burne has, and we believe with justice, rejected this opinion, and has shown that the abscess in question is a circumscribed abscess, within the peritoneum, occasioned in most cases by perforation of the vermiform appendix. Dr. Burne states that he has never met with a case in which the abscess arose from perforation of the cæcum. Such cases are, no doubt, extremely rare. In chronic dysentery, in which there is extensive ulceration of the mucous coat of the large intestine, the submucous cellular tissue is amazingly hypertrophied, so as effectually to prevent perforation. When ulceration of the intestine takes place in the course of an acute disease, as typhoid fever or acute dysentery, there is no thickening of the subjacent coats, and perforation occasionally happens. In typhoid fever, it is generally the lower portion of the ileum that is most diseased, and a perforation there is followed by general peritonitis, which proves speedily fatal: in acute dysentery, on the contrary, the large intestine is the chief seat of disease, and when its coats are destroyed in that part of the cæcum which is not covered by peritoneum, an abscess forms in the cellular tissue behind. We are not prepared to say that such cases occur frequently, even in acute dysentery, but that they do occasionally occur is proved by the following instance:

A man, aged fifty-one, previously in good health, was seized, without obvious cause, with griping, almost incessant purging, and all the symptoms of acute dysentery. At the end of ten days, when we first saw him, a tumour could be felt in the right iliac fossa, nearly as large as the palm of the hand, and exquisitely tender on pressure. He was treated by bleeding, leeches, effervescing draughts. Very little change took place in the tumour, and the dysentery continued unmitigated until five days from our first seeing him, fifteen days from the onset of the complaint, when he was seized suddenly with pain in the abdomen, which was so violent as to draw piercing cries from him, and which was speedily followed by vomiting and collapse. He died at the end of a few hours. The body was examined twenty hours after death. There was considerable sloughing of the mucous coat of the large intestine in a great portion of its extent, and at the termination of the ascending portion all the coats were destroyed, and perforation had taken place. It was this perforation that was the immediate cause of death. In the cæcum also, where it was not covered by peritoneum, all the coats had been destroyed, and there was an infiltration of pus in the cellular tissue behind.

Although, therefore, we are forced to admit that an abscess may form in the right iliac fossa in dependence on the cæcum, from perforation of this gut, yet in an immense proportion of cases it is unquestionably a consequence of perforation of the vermiform appendix. This appendix, from its open mouth terminating in the cæcum, is peculiarly liable to the intrusion of small, hard, undigested substances, which can only escape from it by a retrograde course. If these small bodies remain long in the appendix, and especially if their diameter be large compared with that of the canal, they cause ulceration of its coats, and finally perforation. In the following passage Dr. Burne explains his notion of the manner in which this gives rise to abscess:

"As long as the ulceration is limited to the mucous membrane it is of little consequence, but immediately that the peritoneum is perforated inflammation ensues, for the organic sensibility of the peritoneum will not suffer the presence of any foreign substance. Inflammation, therefore, is lighted up, and may spread with rapidity over the whole continuous surface of this membrane, constituting an universal peritonitis; or the inflammation may be limited to the vicinity of the perforation, may be circumscribed and an abscess form."

Adhesive inflammation of the peritoneum is most probably set up before perforation actually takes place, so that the appendix opens into a space limited by adhesions, and not into the general cavity of the peritoneum. The circumstance that this portion of intestine admits of little motion, and its situation in the iliac fossa, are extremely favorable to the formation of a circumscribed abscess.

The explanation which Dr. Burne has given of the production of these abscesses in the passage we have quoted, is borne out by the details of his cases. The error into which Dupuytren was led respecting their seat and its cause had its origin in the prevailing doctrines of his day, and was not corrected by morbid anatomy. Of the six cases which he has given as cases of abscess in the right iliac fossa, dependent on the cæcum, one only proved fatal: in this case, indeed, the abscess was in the cellular tissue behind the cæcum, but from the details which Dupuytren has given respecting it, we are inclined to believe that it was a case of psoas abscess, originating in caries of the vertebra, and not in disease of the cæcum. (See Leçons Orales. Tom. iii., p. 341.)

We have said that the other cases related by Dupuytren did not prove fatal. A circumstance, however, is mentioned of one of them (obs. 5,) which tends to show that the abscess in that case resulted from perforation of the appendix. After the matter had continued to discharge externally for two or three months, a raisin-stone, which had escaped by the opening, was found in the dressings. Dr. Burne has had more frequent opportunities of making *post-mortem* examinations in such cases. Of the six cases (cases 5, 6, 8, 12, 13, 15,) which he has related, in which there was abscess, four proved fatal. Of these four cases, three presented sloughing of the vermiform appendix; the remaining case (case 12) we believe to be incorrectly classed with the others. With the exception of this case, all those given by Dr. Burne, in which there was abscess in the right iliac fossa in connexion with the cæcum, and which proved fatal, were found by dissection to be cases of circumscribed abscess within the peritoneum, apparently resulting from perforation of the vermiform appendix.

In the course of little more than a year, three cases of abscess in the right iliac fossa, in dependence on the cæcum, have occurred to ourselves. Two of these cases proved fatal; in the third, the abscess opened externally, and, after protracted suffering and the copious discharge of pus having a fecal odour,* the patient recovered. In each of the two fatal cases, there was, as in the cases by Dr. Burne, a circumscribed abscess in the peritoneal cavity, and perforation of the appendix. In one of these cases, the perforation resulted from the presence of an intestinal concretion, of the size of a cherry-stone; in the other, we could discover no such cause for the perforation, which was near the blind extremity of the appendix, and of the diameter of a split pea.

The same conditions, then, being found in all the fatal cases, it seems fair to infer that they existed also in the cases which terminated favorably, and which during life presented the same characters; in other words, that those abscesses which occur in the right iliac fossa, and which are dependent on the cæcum, are not, as Dupuytren imagined, abscesses formed in the cellular tissue, in which the cæcum is imbedded, and sympathetic of irritation of the mucous membrane of that gut, but that they are, primarily, circumscribed abscesses in the cavity of the peritoneum, resulting from perforation of the vermiform appendix.

The cause of this perforation we believe to be, in most cases, the presence of some foreign body, which has become accidentally impacted in the appendix, and which produces sloughing or ulceration of the part beyond it, by causing strangulation, and thus preventing the nutrition

* We infer from this circumstance that the abscess communicated with the cæcum. The intestinal gases do not pass through the coats of the intestines, unless when the latter are dead or strangulated. Fecal odour in the pus, evacuated *during life*, is then, we conceive, indicative of perforation, or, at least, of death or strangulation of the coats of the intestine. In this opinion we differ from M. Grisolle. He says that the pus discharged by an external opening presented a fetid, stercoral odour, in two of his cases, in which there was no reason to believe that the abscess communicated with the intestine. Both of these patients recovered; so that M. Grisolle had no opportunity of examining the condition of the intestine.

of that part. In the cases of Dr. Burne, as well as in those which have fallen under our own notice, the mucous membrane of the large intestine was perfectly sound; so that the ulceration of the appendix was not connected with any general disease of the intestine. In one of the cases that came under our own care, the perforation was *ascertained* to be owing to the presence of a concretion; in two of the cases of Dr. Burne, the extremity of the appendix had sloughed away—a circumstance more likely to result from such a cause than from a simple ulcerative process. The fact that no foreign body was found in these cases, does not form a very strong objection to this reasoning. Every one who has had experience in opening bodies, will confess how readily a small concretion—and the concretion in these cases must necessarily be small—may be overlooked in a large collection of pus, and in the midst of such extensive disease; especially, when the person making the examination does not expect to find it. As a further confirmation of the view we have taken, we may refer to two other cases of Dr. Burne (cases 7, 14), in which there was no abscess, but perforation of the appendix, followed by general peritonitis; and in each of which the perforation was ascertained to have been caused by an intestinal concretion.*

The cases which we have thus grouped together resemble each other in all their essential characters. They occurred for the most part in persons who had suffered no previous disease or derangement of the bowels. The patient was seized with fixed pain in the right iliac fossa, which was soon followed by feverishness, great thirst, a small frequent pulse, anxiety, and, in most cases, by vomiting and by constipation. Soon after the appearance of these symptoms, a deep-seated tumour was felt in the right iliac fossa, at first small, but gradually increasing in size. In some instances the bowels were readily acted upon, in others not; but in neither case did any marked relief follow their evacuation. The abscess, once formed, may either open into the cæcum, in which case the matter becomes discharged by the intestines (Dupuytren, obs. 4, 6; Dr. Burne, case 13); or it may open externally (Dupuytren, 3, 5; Dr. Burne, 5). These terminations appear to be of nearly equal frequency, but the former is the more favorable, from its offering a readier issue to the matter of the abscess, and consequently rendering the suppuration less protracted.

The tumour, when first perceived, has not always precisely the same seat; its situation varying with that of the appendix, which is far from constant. Sometimes it is found in the pelvis; at other times turned upwards, either extended or curved, under the origin of the large intestine.†

*Diagnosis.* The right discrimination of the two classes of cases of tumours in the right iliac fossa, according as they result from an accidental accumulation of fæces in the cæcum, or from perforation of the vermiform appendix, is of the utmost importance with reference both to prognosis and treatment. When of the former kind, the malady is

* Dr. Copland, in his Dictionary of Practical Medicine (art. Cæcum), states that he has met with four instances of the same kind.

† Manuel d'Anatomie, par J. F. Meckel, tom. iii., p. 402.

comparatively slight; and although the tumour may not be readily dispersed, and the symptoms may be of alarming nature, there is seldom much real danger. When of the latter kind, the case is always serious, and the issue often for a long time doubtful. It is from having confounded these different kinds, that some authors have been so free to promise a favorable termination to cases of tumour in the right iliac fossa.*

A correct diagnosis is equally essential as regards treatment. The means that must be perseveringly employed in the one case, will exhaust the patient, and may even be the immediate cause of death, in the other.

The situation of the tumour is, in both cases, the same; and, in either case, it may form rapidly in a person of almost any age,† of either sex, and in the previous enjoyment of good health. When, however, the tumour depends on a collection of fæces, it is the very size of the tumour, and the distension and obstruction which it occasions, which are the cause of all the symptoms; and the tumour is already of considerable magnitude when these symptoms occur, and the attention of the patient is directed to the part. When the tumour depends on perforation of the appendix, the pain and uneasiness, and even more alarming symptoms, precede its appearance; and, if the patient be observed early, the tumour, when first discovered, is small and deep-seated. Once that the tumour has formed, in either case, the bowels are in general confined; but when it results from constipation, the patient experiences great relief from their action: when caused by abscess, their free evacuation is followed by little or no amendment.

The history of the early symptoms, then, but especially the amount of relief which follows the evacuation of the bowels, and the quantity and character of the matters discharged, will generally enable us to determine if the tumour results from an accumulation of fæces. But a tumour from psoas abscess may form in the same situation, and may be difficult to distinguish from the abscess which we have described as the result of perforation of the appendix. Dupuytren says, that in psoas abscess "the pus spreads along the psoas and iliacus muscles; it is deposited in a liquid state in the iliac fossa, and the tumour to which it gives rise is soft and fluctuating from its first appearance. This remark is sufficient to distinguish these purulent collections from those which we have before described."‡ The character here mentioned will aid us much in forming our diagnosis; but if the view we have taken of one of his cases be correct, it was not sufficient to protect even Dupuytren himself from error. It is proper, however, to remark that, in the case to which we allude, the patient first came under Dupuytren's care when the abscess was on the point of breaking, and consequently when the character which he has described as so important was no longer distinctive.

* Dupuytren says, "The prognosis is not generally very unfavorable, since of sixteen cases, observed in very different circumstances, one only proved fatal."—Leçons Cliniq., tom. iii., p. 349.

† An instance has come under our own notice, in which a tumour in the right iliac fossa, from a collection of fæces, gave rise to very alarming symptoms in a little girl, between seven and eight years of age.

‡ Leçons Cliniq., tom. iii., p. 348.

Another element in diagnosis is the degree of the local pain and tenderness. In abscess from caries of the vertebra this is generally slight; while in circumscribed abscess of the peritoneum it is always very great, and is usually attended with general symptoms indicative of the affection—fever, vomiting, hiccough. In cases still doubtful, we may assist our judgment by a consideration of the habit of the patient, and of the presence or absence of pain, uneasiness, or weakness of the loins which usually precedes for some months the appearance of psoas abscess.

Dr. Albers has described another class of cases, in which an abscess forms in the right iliac fossa, from idiopathic inflammation of the cellular tissue in which the cæcum is imbedded (*perityphlitis*). We are inclined to believe that Dr. Albers has taken a wrong view of the cases which he has grouped under this head. In one case, which he has quoted from Puchelt as an instance of this kind, there was caries of the iliac bone! It seems, indeed, that in many of the cases which Dr. Albers has imagined to be cases of idiopathic inflammation of the cellular tissue, there was a similar combination, for he speaks of disease of the bones as a common consequence of suppuration of the cellular tissue. We need hardly remark, that in all probability disease of the bone was the cause and not the consequence of the abscess.

Dr. Albers also notices perforation of the vermiform appendix, as a common termination of abscesses having their origin in the cellular tissue of the iliac fossa. He seems much puzzled to explain this predilection for the appendix; since, as he justly observes, in this affection the cæcum is more exposed to the causes of perforation than the appendix. It would, indeed, be strange if the matter of an abscess, which could find its way through loose cellular tissue to the skin, or to that part of the cæcum which is not covered by peritoneum, were to open into the peritoneal cavity, and subsequently to perforate the peritoneal and other coats of a small floating body like the appendix! Our readers will agree with us that here, as in the instances in which he found a bone diseased, Dr. Albers read the cases backwards, and took for the effect of the abscess what was, in reality, its cause. Dr. Albers has, indeed, given the details of two cases, in which he found an extensive abscess in the cellular tissue of the right iliac fossa, and in which neither perforation of the appendix or cæcum, nor caries of a bone, is noticed.

But in these cases no mention is made of the vertebra, and it does not appear that they were even looked at. Until this omission be supplied, we cannot admit that in these cases the abscess in the iliac fossa was primary: it seems to us much more probable that it was the consequence of disease of the vertebra. In all such cases which have fallen under our own observation, we have been able to trace the matter to this source. The extent in which the bone is diseased is, indeed, often small, bearing no proportion to the size of the abscess; a circumstance which, together with the insidious manner in which scrofulous disease of the vertebra comes on, is sufficient to account for the real origin of the abscess being so often overlooked. We cannot, then, recognize the disease which Dr. Albers has designated *perityphlitis*. An abscess in the cellular tissue of the right iliac fossa, when it does not result from a blow or external wound, from perforation of the appendix or cæcum, or from child-birth, is, we believe, in almost every instance, the consequence of disease of the bones either of the back or pelvis. There may be cases in which an abscess forms in the iliac fossa from idiopathic inflammation of the cellular tissue, but we have never seen one.

*Treatment.*—The nature of the case once ascertained, we can proceed with confidence in our treatment. When the tumour is formed by a collection of fæces, our main reliance must, of course, be on purgatives. If there be considerable pain and tenderness, leeches and fomentations will procure great relief, and may promote the action of other means; but it is only by removing the obstruction that we cure the patient. If powerful cathartics fail of effect, or if they bring on vomiting, we may have recourse to milder ones—to small and repeated doses of salts, which, if the stomach be very irritable, may be given in effervescence. These, partly perhaps from the solvent power of the liquid in which they are given, have often better effect than medicines of more drastic kind. If the obstruction be complete, and symptoms of ileus occur, we have a further resource in bloodletting, and in enemas containing tartar emetic or tobacco.

When the tumour results from perforation of the appendix, we must be careful that we do not, by rough handling or by violent purgatives, destroy the newly-formed adhesions, and thus give rise to general peritonitis. If the fever run high, or the patient be plethoric, we may take blood from the arm, with the view of lessening the inflammation; but we must not waste the patient's strength by repeated bleedings, in the hope of preventing the formation of abscess. Do what we will, the abscess will form, and will eventually discharge itself either externally or into the cæcum. The patient has, at least, a long illness before him, and will require all his strength to support it. A means which we may use with more freedom, and which is more efficacious in relieving the pain and tenderness, is the application of leeches. Besides these, we may prescribe soda-water or effervescing draughts to allay thirst and appease the irritability of the stomach, and wait with patience the issue of the abscess.

In the course of our remarks, we have often referred to the opinion of Dupuytren, that an abscess may form in the cellular tissue behind the cæcum, *sympathetic* of some irritation of the mucous membrane of the neighbouring intestine. In giving this explanation of the origin of the abscesses in question, Dupuytren merely applied to a particular instance a general proposition of Broussais's—a proposition which he considered as one of the key-stones of pathology—that, "when a local irritation reaches a certain height, it repeats itself in other systems or in other organs, and always without change of nature." In his next proposition, Broussais states that it is by means of the nerves that this repetition is effected. "The nerves are the only agents of the transmission of irritation which constitutes morbid sympathies." Since the time when Broussais first promulgated this opinion, greater attention has been paid to the vascular system, as an agent in the dissemination of disease; and every one will now confess that the proposition referred to must lose much of its generality before it will afford a rigorous explanation of facts. A local irritation may, as Broussais seems to have imagined, be transmitted to the brain or spinal cord, and be thence reflected into sensitive or motor nerves, so as to produce pain or muscular spasm in a part remote from the original seat of disease: pain of the knee from diseased hip; of the testicle from the passage of a calculus along the ureter; of the shoulder from disease of the liver; cramps of the legs from the presence of worms in the intestinal canal; convulsions from teething,—may all be adduced as familiar examples of such *sympathetic* affections. But we question if a well-authenticated instance can be cited of an abscess produced in this manner. Abscesses that have been called *sympathetic* generally form in the course of the lymphatics leading from the part primarily affected, and result from inflammation propagated along those lymphatics, or, perhaps more frequently, from the absorption of some irritating matter. But if abscesses ever do form in the way that Broussais imagined,* we certainly have no evidence that abscesses in the cellular tissue behind the cæcum result from irritation, however great or prolonged, of the mucous membrane of the intestine. Numerous cases of dysentery, of years' standing, have fallen under our notice, in which the mucous membrane of the cæcum was destroyed in a great extent, and the submucous cellular tissue thickened and almost cartilaginous, yet we have never met with an abscess in the cellular tissue behind, which we could ascribe to irritation of the lining membrane of the gut. We are happy to find that on this point, as on many others, our opinion is borne out by the experience of Dr. Burne.

*Statistics.*—The view taken of these affections by M. Grisolle, in his "History of Phlegmonous Tumours in the Iliac Fossæ," is more comprehensive than that of Dr. Burne or Dr. Albers; as he includes all phlegmonous tumours which appear in either fossæ, whether they result from disease of the intestines, from child-birth, or from any other cause. The memoir is very elaborate, and consists principally of an analysis of seventy-three cases, twelve only of which came under the observation of M. Grisolle *himself:* the rest he collected from different sources. It is from these cases that, to use his own words, he attempts to solve all problems connected with the history of this disease. In fifty-three of these seventy-three cases, the abscess was in the right iliac fossa; and in twenty in the left. Seventeen of the cases occurred soon after child-birth; the rest were independent of the puerperal state. Nine of the seventy-three cases terminated in complete resolution, and in nine others resolution had commenced; but the engorgement had not entirely disappeared when the patients left the hospital; so that in eighteen of the cases the only evidence of abscess was derived from the general symptoms and from the existence of a tumour.

We are much inclined to doubt the truth of the opinion that abscesses and inflammatory engorgements of the cellular tissue are so frequently resolved; and believe that in most of the cases said to have terminated

* Broussais divided morbid sympathies into two kinds: the first manifesting itself by organic phenomena, as congestions, alterations of secretions, increase or diminution of exhalation or absorption, change of temperature, faults ot nutrition; the second by pain, by convulsive movements of the voluntary muscles, by mental aberration. The former he designated organic sympathies; the latter sympathies of relation. He supposed that both were exercised solely by means of the nervous system, but that organic sympathies might take place through the great sympathetic; whereas sympathies of relation required the intervention of the brain and spinal marrow. See prop. 86, et seq.

in this manner the tumour in the iliac fossa resulted simply from a collection of fæces, especially as M. Grisolle, in a subsequent part of his paper, tells us that resolution is very rare when the treatment is commenced later than the fifth or sixth day from the appearance of the earliest symptoms.

Notwithstanding the variety in the cause of these abscesses, and their different terminations, M. Grisolle proceeds with his analysis, as if the cases were all alike. When treating of symptoms, he tells us in how many of the cases there was pain in the iliac fossa at the commencement of the disease, in how many not; that diarrhœa preceded the appearance of the tumour in one twelfth of the cases, constipation in one tenth, alternations of diarrhœa and constipation in rather less than a twentieth, &c.; and, in like manner, when speaking of treatment, he informs us that bleeding was freely practised in twenty of the cases, and enters into a laboured investigation of the effect which this produced, without any distinction as to the kind of cases to which this treatment was applied.

Although we cannot too much commend the industry of M. Grisolle in collecting his materials, we cannot but think such an analysis of them very valueless, and a strange abuse of the numerical method. What is the use of calculations of this nature applied without discrimination to cases of abscess resulting from such a variety of causes—from perforation of the appendix; from childbirth; from blows; and, perhaps, from caries of the vertebræ? A much more profitable task would have been to have grouped together those cases which had clearly the same origin, and to have analyzed them, with the view of discovering in what characters they differed from other cases having with them some points of resemblance, but essentially different in kind; and thus affording us some guidance in arriving at a correct diagnosis of an individual case.

The very abundance of the materials which M. Grisolle has accumulated serves also to increase the disorder. He tells us, at the commencement of his paper, that a great number of the cases which he found recorded in the Archives of Science were incomplete, and wanting in important details. It would have been much wiser to have discarded these cases, which only obscure the light given by the others. The numbers analyzed would, indeed, have been smaller; and the paper would not have had the statistical air which it has at present: but this defect would have been more than compensated by greater clearness and precision.

Approving much, as we do, of the numerical method when legitimately used,—that is, in the proper cases and in the proper manner,—we cannot too strongly protest against its abuse. One of these abuses is flagrant in the memoir of M. Grisolle; we mean the neglect of carefully selecting the materials to which this method is applied. If different objects be confounded, the greater the number of observations, the greater the certainty that the results obtained from them will be incorrect. To borrow an illustration from astronomy—an astronomer, to determine the place of a star, is not satisfied with a single observation, because he knows that his lenses and instruments are not perfect enough to enable him to avoid small errors of observation: he makes a great number of observations; and on the supposition that the errors of individual observations will be some in excess and others in defect, and that many of them will thus compensate and destroy each other, he rightly takes the *mean* place, determined by all his observations, as the place of the star. But suppose that he confounded two stars, and sometimes observed one, and sometimes the other, it is clear that the mean place resulting from his observations would be some place between the two, and would be more certainly incorrect than the place given by a single observation. Or, suppose that he always observed the same star, but that the instrument with which his observations were made had some bias, which tended, for instance, to give an error in defect: this error would be common to all the observations; it would be a constant quantity, and would, therefore, appear in the result with much greater certainty than in any single observation. If the astronomer had no means of ascertaining, and so making allowance for this bias of his instrument, all his calculations would be vitiated, and, however great the number of observations, the calculated place of the star would not be the true place, but the true place diminished by the mean error caused by the bias of the instrument.

It is the same when statistics are applied to medical subjects. If different objects be confounded, or if the observer have a particular bias, the conclusion from a great number of observations will be more certainly erroneous than that from a single one. It is this fault of confounding different objects that renders valueless a great part of the calculations of M. Grisolle; and, on account of it, we shall refrain from entering into a complete analysis of his paper, which, nevertheless, contains many judicious remarks.

M. Grisolle agrees with Dr. Burne in rejecting the notion entertained by Dupuytren and others, that abscesses form in the cellular tissue behind the cæcum, *sympathetic* of irritation of the mucous membrane of the intestine. The arguments he makes use of to support his opinion are the same as those employed by Dr. Burne.

Dupuytren states that particular occupations predispose to abscess in the right iliac fossa, and that this complaint is especially common in house-painters, colour-grinders, turners in copper, and persons continually exposed to the dust and emanations from irritating metals. Both Dr. Burne and M. Grisolle have been unable, from a review of their cases, to discover any such influence in the occupations in question. The reason of this disagreement is, that Dupuytren supposed all tumours in the right iliac fossa to be phlegmonous, and consequently reckoned as such fecal tumours, which are no doubt most common in the classes he has specified; while Dr. Burne and M. Grisolle have confined themselves more exclusively to *abscesses* which, as we have seen, rarely result from an accumulation of fæces.

The most interesting observations in the paper of M. Grisolle are those relating to abscesses which form in the iliac fossa after delivery. We are not prepared to enter into the consideration of these cases, as we have wished, in the present article, to confine our attention to abscesses which are dependent on the cæcum, referring to those which arise from other causes only so far as is necessary to illustrate our diagnosis. The circumstance of the appearance of the abscess soon after childbirth is of itself almost sufficient to distinguish those which result from this cause. We shall, therefore, subjoin without comment some of the statements of M. Grisolle respecting these cases.

Of seventeen cases of this kind which he has collected, the abscess was seated in eleven on the left side, and in six on the right. M. Grisolle can assign no reason for the greater frequency of the abscess on the left side. The disease appeared in eleven of the cases from the third to the tenth day after delivery; in two from the tenth to the fifteenth; in one at the end of a month. In the remaining three cases, the date of the appearance of the disease was not specified.

The prognosis is more unfavorable in cases of abscess after childbirth than in others: seven of these seventeen cases, nearly one half, having proved fatal. M. Grisolle says, "A circumstance worthy of notice is, that none of the patients that have fallen under my own observation had suckled their infant; and the same seems to have been the case with almost all the women whose histories have been left us by authors."

